# Psychosocial stressors contributing to emergency psychiatric service utilization in a sample of ethno-culturally diverse clients with psychosis in Toronto

**DOI:** 10.1186/s12888-017-1487-8

**Published:** 2017-09-02

**Authors:** Martin Rotenberg, Andrew Tuck, Kwame McKenzie

**Affiliations:** 1 Department of Psychiatry, University of Toronto, Toronto, ON Canada; 2 Centre for Addiction and Mental Health, 33 Russell Street, Room 2017, Toronto, ON M5S 3B1 Canada

**Keywords:** Psychosis, Ethnicity, Psychosocial stressors, Emergency department

## Abstract

**Background:**

Understanding the psychosocial stressors of people with psychoses from minority ethnic groups may help in the development of culturally appropriate services. This study aimed to compare psychosocial factors associated with attendance at an emergency department (ED) for six ethnic groups. Preventing crises or supporting people better in the community may decrease hospitalization and improve outcomes.

**Method:**

A cohort was created by retrospective case note analysis of people of East-Asian, South-Asian, Black-African, Black-Caribbean, White-North American and White-European origin groups attending a specialized psychiatric ED in Toronto with a diagnosis of psychosis between 2009 and 2011. The psychological or social stressors which were linked to the presentation at the ED that were documented by the attending physicians were collected for this study. Logistic regression models were constructed to analyze the odds of presenting with specific stressors.

**Results:**

Seven hundred sixty-five clients were included in this study. Forty-four percent of the sample did not have a psychiatrist, and 53% did not have a primary care provider. Social environmental stressors were the most frequent psychosocial stressor across all six groups, followed by issues in the primary support group, occupational and housing stressors. When compared to White-North American clients, East-Asian and White-European origin clients were less likely to present with a housing stressor, while Black-African clients had decreased odds of presenting with primary support group stressor. Having a primary care provider or psychiatrist were predominantly protective factors.

**Conclusion:**

In Toronto, moving people with chronic mental health conditions out of poverty, increasing the social safety net and improving access to primary care and community based mental health services may decrease many of the stressors which contribute to ED attendance.

## Background

The Mental Health Commission of Canada argues that people from different ethnic groups in Canada have different service needs. These have not been fully examined but are reflected in differences in service use, pathways to care and pathways to emergency services [1–4]. Differences in exposure to the social determinants of health and specifically psychosocial stressors could underlie differences in need.

Psychosocial stressors are associated with increased symptomatology and relapse in clients with schizophrenia [5]. They increase the risk of admission to hospital. For instance, life events − which are characterized by a sudden change in a person’s social milieu (e.g., death of a relative, breaking up with a partner) − are associated with worsening of symptoms [6]. Traumatic events (e.g., repeated childhood sexual abuse) increase the odds of hospitalization [7]. Furthermore, there is a considerable body of research on expressed emotion (EE) as a specific psychosocial stressor. EE is characterized by high levels of criticism, hostility, and emotional over involvement within the family unit, and is a significant risk factor predicting relapse in clients with schizophrenia [8]. The importance of psychosocial stressors is underlined by their presence in the five axial formulation of the DSM-IV [9]. Understanding psychosocial stressors is considered fundamental to assessment and the planning of care.

There is a growing international literature investigating psychosocial stressors in minority ethnic communities. Studies of clients of African-Caribbean origin in the United Kingdom, and clients of Moroccan origin in Belgium suggest that unemployment and interactions with social supports may be an important psychosocial risk factor for hospitalization [10, 11].

Toronto, Ontario is the largest city in Canada and one of the most diverse cities in the world. Half of the population are first generation immigrants [12]. Differential patterns of service use such as an increased use of emergency services and coercive measures under the Mental Health Act have been reported in some ethnic groups with psychosis during times of crisis [4]. There has been little examination of the role of psychosocial stressors in such disparities.

Because stressors play a role in relapse and admission, initiatives that reduce specific stressors could improve clinical and functional outcomes [13]. A closer examination of differences in stressors that clients of specific ethnic groups face may help develop services that more appropriately meet their needs. A better understanding of the causes of differences in care and outcomes is considered fundamental to the development of equitable services by the Mental Health Commission of Canada [14].

This study aimed to investigate the psychosocial factors that were considered by psychiatrists to have led to the deterioration in mental state and presentation of clients with psychosis to a psychiatric emergency department (ED) in Toronto. Six different ethnic groups were compared. We wanted to investigate whether psychosocial stressors contributing to a crisis or ED presentation in patients with psychosis differed by ethnic group. The overall objective was to better understand factors that led to presentation as a pre-requisite for the development of more appropriate service interventions.

## Methods

### Study design

A retrospective chart review was conducted on clients who presented to the ED between the years 2009–2011. Clients included in the study were part of a cohort built by purposefully sampling clients who attended a stand-alone psychiatric ED in Toronto, Canada from each of the six largest ethnic group in Toronto, (East-Asian, South-Asian, Black-Caribbean, Black-African, White-North American, White-Canadian), for each of the years 2009, 2010, 2011. The final study population was 765 because in some years there were less than the targeted goal of 50 clients with psychosis who attended the ED from some of the ethnic groups.

A standardized assessment form is completed for all clients who attend the ED by the multi-disciplinary clinical team including a psychiatrist, social worker and nurse. The assessment form contains, socio-demographic data, clinical data including symptoms, history and diagnosis as well as questions on what led to presentation to the ED. Current psychosocial stressors are documented by physicians using the DSM-IV-TR Axis IV diagnosis.

For full details on the sampling methodology see Rotenberg et al. 2017 [4]. A number of factors shown to be linked to type of presentation were collected for this study [1].

### Measures

#### Socio-demographic data

Data on age and gender were collected.

#### Ethnicity

Ethnicity was recorded as per client’s self-identified ethnicity using 18 categories developed by the Toronto District School Board which are commonly used in Ontario [15].

#### GP and psychiatrist

Data was recorded as to whether clients have a primary care provider and psychiatrist in the community.

#### Severity of psychosis

An index of psychosis severity was developed based on that reported by Jarvis et al. 2005 [1]. We used data that was systematically collected during the chart review. One point was scored (up to a maximum of 14) for the presence of each of the following via mental status examination and clinical history: hallucinations, delusions, ideas of reference, evidence of thought disorder, bizarre behavior, chart diagnosis of schizophrenia or schizoaffective disorder, previous psychiatric admission, evidence of self-neglect, guarded/suspicious/uncooperative with clinical interview, poor insight, poor judgment, poor impulse control, aggressive behavior, agitation.

#### Psychosocial stressors

Information pertaining to psychosocial stressors which contributed to an individual’s ED presentation was available in client charts under the DSM-IV-TR Axis IV diagnosis heading. Stressors were coded and categorized into the 9 DSM-IV-TR Axis IV categories, with an additional category constructed if no psychosocial stressors were indicated in the chart. The Axis IV categories utilized with examples of specific stressors:

1. Problems with primary support group − family discord, child and marital issues, conflict with parents, death in the family, sexual or physical abuse, child apprehended by child services
2. Problems related to the social environment − socially isolated, no friends, living alone, no social supports, manipulated by peers, challenges with peer groups
3. Educational problems − academic problems, stress from education program
4. Occupational problems − unemployed, stress at work, workplace disputes
5. Housing problems − homelessness, underhoused, no fixed address, stress due to housing situation
6. Economic problems − financial difficulties, poverty, issues with disability support payments
7. Problems with access to health care − health care provider not accessible, no health provider, no health insurance
8. Problems related to interaction with the legal system/crime − legal charges, court appearance, recent release from jail, probation
9. Other psychosocial and environmental problems − any issues related to immigration that were not social or occupational in nature, including refugee status & refugee claimant process.

Charts that had information that was illegible were coded as none indicated. The number of psychosocial stressors on presentation was also recorded. Two authors (MR, AT) came to agreement on the coding of all stressors.

### Statistical analysis

All statistical analyses for this study were conducted in SPSS 21.0 [16]. Due to the infrequency of the following categories in the sample − i) problems with access to healthcare services, ii) problems associated with the legal system, and iii) other psychosocial and environmental problems − they were collapsed into a single category labeled as other psychosocial stressors to ensure sufficient power for analyses. Univariate analyses (Chi-square, ANOVA) were conducted to determine if there were any differences between the ethnic groups at baseline and the relationship between predictor variables with the outcome variables (the psychosocial stressor categories). Binary logistic regression models were constructed to examine the relationship between the psychosocial stressor categories that contributed to the ED presentation and the predictor variables (demographic, ethnicity, GP, psychiatrist and symptom severity). Individual regression models were constructed to predict the outcome psychosocial stressor category, however only psychosocial stressor categories that were sufficiently powered (*n* > 100*)* were utilized as outcome variables. Variables were included in the regression models as predictors only if there was an association with the outcome variables via univariate analyses with a *p*-value of < 0.1 or had been previously reported in the literature. All means are reported with a standard deviation, and all odds ratios are reported with a 95% confidence interval.

### Ethics

This study obtained ethics approval from the Centre for Addiction and Mental Health’s research ethics board.

## Results

### Sample characteristics

Of the 765 clients included in the study there were no statistically significant differences in diagnoses among the ethnic groups (Χ^*2*^ = 25.243, *df* = 20, *p* = 0.192). The majority of clients were diagnosed with schizophrenia (47.6%), followed by schizoaffective disorder (14.4%), Psychosis NOS/NYD (22%), a combination of diagnoses of which one included schizophrenia or schizoaffective disorder (8.4%), and all other diagnoses (7.7%) including brief psychotic episode, schizophreniform disorder, brief psychotic episode, first episode psychosis and substance induced psychosis.

The mean age of the sample was 38.01 ± 14.13, with a significance age difference between the ethnic groups, *F*(5, 754) = 3.676, *p* < 0.01 with the Black-African (33.45 ± 11.076) and South-Asian (35.73 ± 11.934) groups being younger.

Only 55.7% of the sample had a psychiatrist, and 46.8% had a GP. Between ethnic groups there were no differences in having a psychiatrist (Χ^*2*^ = 4.452, *df* = 5, *p* = 0.486) and GP (Χ^*2*^ = 4.413, *df* = 5, *p* = 0.492).

### Psychosocial stressors

A descriptive profile of clients by psychosocial stressor is presented in Table 1. The mean number of psychosocial stressors on presentation was 1.37 ± 1.08, with a range of 0–5. There were no significant difference between the ethnic groups and number of stressors on presentation, *F*(5, 759) = 1.49, *p* = 0.19. The most frequent stressors on presentation in this sample were social environmental stressors (35.2%, *n* = 269), followed by primary support group (24.1%, *n* = 184), occupational (19.7%, *n* = 151), housing (18.8%, *n* = 144), economic (14.9%, *n* = 114), other (11.9%, *n* = 91), and educational (5.1%, *n* = 39) stressors. Of the 175 stressors categorized as none indicated only five were categorized as such for either being illegible or marked with a question mark. The majorities of the none indicated category were left blank, or were medical or psychiatric issues, rather than psychosocial ones. Forty one percent of clients had more than one psychosocial stressor, 37% had only one psychosocial stressor and 22% of the sample did not have a stressor indicated in the chart.

**Table 1:** Descriptive profile of emergency department clients with diagnosis of psychosis by psychosocial stressor (*n* = 765)

Variable	Stressor: housing (*n* = 144)	Stressor: primary support group (*n* = 184)	Stressor: social environment (*n* = 269)	Stressor: economic (*n* = 114)	Stressor: occupation (*n* = 151)	Stressor: education (*n* = 39)	Stressor: other (*n* = 91)	Stressor: none indicated (*n* = 173)
East-Asian (*n* = 137)	16 (11.1%)	37 (20.1%)	49 (18.2%)	10 (8.8%)	20 (13.2%)	11 (28.2%)	11 (12.1%)	40 (23.1%)
South-Asian (*n* = 91)	20 (13.9%)	29 (15.8%)	32 (11.9%)	14 (12.3%)	21 (13.9%)	8 (20.5%)	14 (15.4%)	9 (5.2%)
Black-African (*n* = 99)	19 (13.2%)	10 (5.4%)	32 (11.9%)	19 (16.7%)	23 (15.2%)	6 (15.4%)	19 (20.9%)	21 (12.1%)
Black-Caribbean (*n* = 141)	33 (22.9%)	27 (14.7%)	52 (19.3%)	32 (28.1%)	24 (15.9%)	4 (10.3%)	18 (19.8%)	26 (15.0%)
White-North American (*n* = 150)	37 (25.7%)	35 (19.0%)	53 (19.7%)	22 (19.3%)	29 (19.2%)	5 (12.8%)	18 (19.8%)	37 (21.4%)
White-European (*n* = 147)	19 (13.2%)	46 (25.0%)	51 (19.0%)	17 (14.9%)	34 (22.5%)	5 (12.8%)	11 (12.1%)	40 (23.1%)
Male	91 (64.1%)	110 (61.1%)	98 (63.0%)	66 (58.4%)	111 (74.0%)	31 (79.5%)	67 (75.3%)	95 (57.2%)
Age w/ Stressor (Mean ± SD)	38.48 ± 12.83	36.30 ± 14.44	39.26 ± 14.31	39.43 ± 12.74	33.07 ± 10.59	23.15 ± 4.37	34.63 ± 12.47	40.60 ± 14.32
Age w/o Stressor (Mean ± SD)	37.94 ± 14.43	38.56 ± 14.00	37.34 ± 14.00	37.77 ± 14.35	39.22 ± 14.62	38.82 ± 14.03	38.48 ± 14.29	37.26 ± 14.00
GP	48 (34.3%)	99 (54.7%)	112 (42.1%)	49 (44.1%)	76 (51.0%)	24 (61.5%)	41 (45.6%)	84 (50.3%)
Psychiatrist	79 (56.8%)	97 (53.3%)	133 (50.0%)	51 (45.9%)	71 (47.3%)	25 (64.1%)	42 (46.7%)	109 (64.9%)
Symptom Severity Scale w/ Stressor Indicated (Mean ± SD)	5.33 ± 2.43	4.38 ± 2.36	5.07 ± 2.55	5.33 ± 2.38	4.27 ± 2.60	3.74 ± 2.35	5.04 ± 2.60	5.23 ± 2.56
Symptom Severity Scale w/o Stressor Indicated (Mean ± SD)	4.70 ± 2.55	4.96 ± 2.58	4.68 ± 2.52	2.55 ± 4.73	4.95 ± 2.50	4.88 ± 2.54	4.79 ± 2.53	4.70 ± 2.52

## Regression models

Table 2 presents the models predicting each of the psychosocial stressor outcome categories that had more than 100 observations in the sample.

**Table 2:** Predictors of psychosocial stressors among emergency department clients (*n* = 730)

Variable	Model 1 housing	Model 2 primary support group	Model 3 social environment	Model 4 economic	Model 5 occupation	Model 7 none indicated
East-Asian	0.35 (0.18–0.70)**	1.14 (0.66–2.00)	1.11 (0.67–1.84)	0.49 (0.22–1.10)	0.68 (0.35–1.29)	1.39 (0.80–2.42)
South-Asian	0.88 (0.46–1.68)	1.29 (0.70–2.36)	1.07 (0.61–1.88)	1.03 (0.48–2.20)	0.96 (0.50–1.86)	0.41 (0.19–0.92)*
Black-African	0.72 (0.38–1.38)	0.36 (0.17–0.79)*	0.88 (0.50–1.55)	1.43 (0.71–2.91)	0.99 (0.51–1.90)	0.93 (0.49–1.77)
Black-Caribbean	0.88 (0.50–1.54)	0.83 (0.46–1.49)	1.11 (0.68–1.83)	1.56 (0.83–2.92)	0.88 (0.47–1.63)	0.65 (0.35–1.19)
White-European	0.50 (0.27–0.92)*	1.42 (0.83–2.43)	1.01 (0.61–1.66)	0.82 (0.41–1.65)	1.10 (0.61–1.97)	1.22 (0.71–2.12)
Age	1.00 (0.99–1.02)	0.99 (0.97–1.00)*	1.01 (0.99–1.02)	1.01 (0.99–1.02)	0.97 (0.96–0.99)***	1.01 (1.00–1.03)
Male	0.96 (0.64–1.44)	0.80 (0.55–1.15)	1.07 (0.77–1.49)	0.80 (0.52–1.24)	1.58 (1.03–2.40)*	0.77 (0.53–1.12)
Symptom Severity Scale	1.08 (1.00–1.16)*	0.94 (0.88–1.01)	1.06 (0.99–1.31)	1.07 (0.98–1.16)	0.94 (0.87–1.02)	1.07 (0.99–1.15)
GP	0.55 (0.37–0.81)**	1.32 (0.93–1.88)	0.73 (0.53–0.99)*	0.87 (0.57–1.33)	1.19 (0.81–1.74)	1.22 (0.84–1.76)
Psychiatrist	1.00 (0.68–1.47)	0.86 (0.61–1.23)	0.64 (0.47–0.88)**	0.59 (0.39–0.90)*	0.69 (0.47–1.01)	1.56 (1.07–2.28)*
Constant	0.29**	0.77	0.40*	0.15***	0.83	0.12***
Χ^2^	31.97***	32.63***	20.93*	25.94**	38.88***	33.26***
df	10	10	10	10	10	10

Reference Categories: ethnicity (White-North American); gender (female); GP (no GP); Psychiatrist (no Psychiatrist)

Significance: *p < 0.05, **p < 0.01, ***p < 0

### Ethnicity

East-Asian (0.35 OR, 95% CI 0.18–0.70) and White-European (0.50 OR, 95% CI 0.27–0.92) clients have decreased odds of presenting with housing stressors when compared to White-North American clients. Black-African clients are 64% less likely (0.36 OR, 95% CI 0.17–0.79) to present with a primary support group stressor when compared to White-North American clients. South-Asian clients are 59% less likely (0.41 OR, 95% CI 0.19–0.92) to present without any psychosocial stressors indicated in the ED chart when compared to White-North American clients.

### GP and psychiatrist

Having a GP decreases the odds of presenting with a housing stressor and social environment stressor. Having a psychiatrist decreases the odds of presenting with a social environmental stressors, economic stressor and other stressor, when compared to clients without a psychiatrist. Clients with a psychiatrist are 1.56 times more likely (95% CI 1.07–2.28) to present without any psychosocial stressor indicated in the ED chart.

### Age and gender

With increasing age the odds of presenting with a primary support group (0.99 OR, 95% CI 0.97–1.00), or occupational (0.97 OR, 95% CI 0.96–0.99) stressor are all decreased. Male clients are 1.56 times more likely (95% CI 1.03–2.40) to present with an occupational stressor when compared to female clients.

## Discussion

Our study of 765 clients with psychosis attending a downtown psychiatric ED in a high-income country with universal health coverage found that access to health services in the community and psychosocial stressors were prominent factors in their presentation. 44.3% of the clients presenting to the ED with psychosis did not have a psychiatrist and 53.2% did not have a GP. Forty one percent of clients had more than one psychosocial stressor. Across all ethnic groups, social environmental stressors followed by primary support group, occupational and housing stressors were the most frequent contributing stressors in ED attendance. There were more similarities than differences in psychosocial stressors in our cohort.

### Limitations and strengths

A major strength of this study is the sample size. The large number of clients included in the analysis allows for comparison across several ethnic groups. Data that was utilized in this study was routinely collected by clinicians, therefore the sample was not biased by the fact the study was underway. Limitations to this study include, that as a retrospective study only associations can be drawn, that there may be variation in how clinicians rate clinically significant stressors [17], and that clinicians may have difficulty judging the significance of stressors [18]. Furthermore, rating of stressors may rely on a clinician’s perception and interactions between clinician and client ethnicity may exist and can influence how psychosocial stressors are rated [19], which due to methodological constraints cannot be accounted for in this study. Within group differences and variance is also an important factor to consider and was not a focus of this study. Furthermore, since the DSM-V no longer utilizes a multi-axial diagnostic system it may be challenging to reproduce the outcome measures in future studies.

### Interpretation of results

There is a dearth of research on the impact of psychosocial factors on the outcome of psychosis in minority ethnic group populations in Canada, and how such stressors may differ between groups. Our study found that regardless of ethnicity, social environment stressors (e.g., social isolation, and lack of social supports) are the most prominent psychosocial factor contributing to ED presentations in all clients. This was followed by primary support group, occupational, and housing stressors.

There appears to be more similarities than differences between ethnic groups with regards to psychosocial stressors. Having a primary care provider and psychiatrist in the community are both important protective factors. Yet, only half of the clients have access to health care providers in the community, regardless of their ethnic group. The lack of differences between groups may be due to the lack of primary and mental health care being provided in the community, resulting in all clients presenting to the ED for services.

The frequency of social environmental and primary support group stressors, along with occupational stressors in this study, highlights the importance and need for psychosocial rehabilitation supports. Evidence based psychosocial rehabilitation interventions that can take place in the community, such as skills training, family psychoeducation and supported employment, are all effective at reducing hospitalization and the overall cost of care [20].

In Toronto racialised groups are at a higher risk of being precariously housed [21], a recent study has found that racialized clients with psychosis who are precariously housed may experience additive stress from racism, episodes of homelessness and the stigma of mental illness [22]. Our study found that clients from East-Asian and White-European ethnic groups may be less likely to present to the ED due to a housing stressor. In this sample the majority of housing stressors were related to homelessness. It may be that physicians were more likely to document homelessness and missed stressors associated with being precariously housed. Future research may benefit from a more detailed analysis of both housing needs and stressors individuals may face.

Overall this study found more similarities than differences in stressors impacting presentation to the ED for clients of diverse ethnic backgrounds with psychosis. Any association between the impact of ethnic differences in psychosocial stressors and presentation to ED may have been drowned out by the weight of social difficulties to which all people with psychosis in Toronto are exposed to and because so many of the clients did not have access to primary care and a psychiatrist.

Health care, including psychiatric care, is publicly funded in Toronto. There is a range of services available for people with psychoses, including assertive community treatment teams, intensive case management teams, case management teams, supportive housing, outpatient psychiatric care and acute care hospital beds. Access to these services may not be equitable. The gateway to medical and mental health services, as well as an important pathway to social services is via primary care providers. In Ontario, GPs in the primary care setting provide most of the mental health care to individuals with mental health conditions, including ongoing care to individuals with severe and persistent conditions like schizophrenia. Psychiatrists, who provide specialized care, also facilitate access to services that may be beneficial to the recovery and ongoing care of individuals with psychosis in the community.

Most clients with a severe and persistent mental illness may be eligible to receive disability support which provides financial assistance, as well as other benefits and services. For an individual to apply for government disability assistance an application must be completed in conjunction with a physician. If an application is successful, individuals will still be living below the poverty line, receiving between $20–35 a day, with housing costs accounting for 70–90% of such allowances [23]. It may not, therefore be surprising that social, occupational and housing stressors all have a significant impact on clients in this sample. Increased investment in the social safety net, access to primary care and community mental health supports may help to decrease the wide variety of stressors which contribute to attending the ED for care and the potential for a costly inpatient admission.

Acute hospital and inpatient care for mental health issues are the second most costly admissions in the Canadian healthcare system [24]. Developing equitable primary care services, mental health care services and social services, which are accessible by clients of all backgrounds, so that there is a decreased use of hospital inpatient beds and emergency departments, is one aim of a high functioning mental health service and system.

## Conclusion

Approximately half of the clients presenting to the ED with psychosis do not have a psychiatrist or a GP, yet having a health care provider in the community reduces the odds of presenting to the ED with a variety of psychosocial stressors. Across all ethnic groups, social environmental stressors followed by primary support group, occupational and housing stressors were the most frequent contributing stressors in ED attendance. East-Asian and White-European origin clients are less likely to present with a housing stressor, while Black-African clients are less likely to present with primary support group stressor when compared to White-North American clients. Pulling people with psychosis out of poverty, and increasing social services and community based mental health supports may help reduce stressors which can contribute to a crises and ED attendance.

## Abbreviations

ED: Emergency department
EE: Expressed emotion
GP: General practitioner
